# Protocadherins, not prototypical: a complex tale of their interactions, expression, and functions

**DOI:** 10.3389/fnmol.2013.00004

**Published:** 2013-03-19

**Authors:** Joshua A. Weiner, James D. Jontes

**Affiliations:** ^1^Department of Biology, The University of IowaIowa City, IA, USA; ^2^Department of Neuroscience, The Ohio State UniversityColumbus, OH, USA

**Keywords:** protocadherins, cadherin superfamily, adhesion, neural circuits, Pcdh

## Abstract

The organization of functional neural circuits requires the precise and coordinated control of cell–cell interactions at nearly all stages of development, including neuronal differentiation, neuronal migration, axon outgrowth, dendrite arborization, and synapse formation and stabilization. This coordination is brought about by the concerted action of a large number of cell surface receptors, whose dynamic regulation enables neurons (and astrocytes) to adopt their proper roles within developing neural circuits. The protocadherins (Pcdhs) comprise a major family of cell surface receptors expressed in the developing vertebrate nervous system whose cellular and developmental roles are only beginning to be elucidated. In this review, we highlight selected recent results in several key areas of Pcdh biology and discuss their implications for our understanding of neural circuit formation and function.

## Introduction

The organization of functional neural circuits requires the precise and coordinated control of cell–cell interactions at nearly all stages of development, including neuronal differentiation, neuronal migration, axon outgrowth, dendrite arborization, and synapse formation and stabilization. This coordination is brought about by the concerted action of a large number of cell surface receptors, whose dynamic regulation enables neurons (and astrocytes) to adopt their proper roles within developing neural circuits. While a large number of protein families have been identified that may play roles in neural circuit formation, detailed cellular functions and, especially, molecular mechanisms have been elucidated for only a handful.

The protocadherins (Pcdhs) comprise a major family of >80 cadherin superfamily molecules expressed primarily in the developing vertebrate nervous system, with lower expression seen in other organs such as lung and kidney. The cadherin superfamily is a diverse collection of cell-surface molecules defined by the presence of several ~110 amino acid extracellular cadherin (EC) motifs (Nollet et al., 2000; Hulpiau and Van Roy, 2009, 2011). The canonical members of this superfamily, the classical cadherins, are type I transmembrane proteins containing 5 EC repeats and a conserved cytoplasmic domain that interacts with the armadillo repeat proteins, β-catenin and p120ctn (Gumbiner, 2005; Takeichi, 2007; Nelson, 2008; Niessen et al., 2011). The classical cadherins mediate calcium-dependent, primarily homophilic, adhesion through interactions between their EC1 domains (N-terminal, most distal from the cell membrane). In a search for additional classical cadherins using degenerate PCR, Suzuki and colleagues discovered a related family of molecules, which they named “Pcdhs” (Sano et al., 1993). The Pcdhs are structurally similar to classical cadherins in that they are also type I transmembrane proteins containing 6 or 7 EC repeats, but their cytoplasmic domains are distinct and lack catenin-binding sites (Sano et al., 1993; Wu and Maniatis, 1999; Nollet et al., 2000; Vanhalst et al., 2005). Subsequently, Pcdhs have been shown to comprise a large and diverse collection of molecules, which are expressed broadly in the developing and mature vertebrate nervous system.

Pcdhs can be divided into two broad classes: the clustered Pcdhs (encoded by the *Pcdh-α*, -*β*, and -*γ* gene clusters, encompassing ~60 genes in mammals) and the non-clustered Pcdhs (so-called δ-Pcdhs) (Hulpiau and Van Roy, 2009, 2011). In mammals, the clustered Pcdh genes lie in three tandem arrays encompassing ~1 MB at human chromosome 5q31 and on mouse chromosome 18 (Wu and Maniatis, 1999; Sugino et al., 2000; Wu et al., 2001). Within the *Pcdh-α* and -*γ* clusters, multiple large “variable” exons encoding 6 EC domains, a transmembrane domain, and a variable cytoplasmic domain are each expressed from their own promoters and spliced to three small “constant” exons that encode a shared C-terminal domain (the *Pcdh-β* locus contains no such “constant” exons) (Tasic et al., 2002; Wang et al., 2002a; see Figure 2B). The largest group of non-clustered Pcdhs consists of the δ1 (7 EC domains) and δ2 (6 EC domains) sub-families, which are distantly related, yet exhibit short, conserved sequence motifs in their cytoplasmic domains (Wolverton and Lalande, 2001; Vanhalst et al., 2005).

The involvement of Pcdhs in neural circuit formation has been inferred on the basis of their structural homology to the classical cadherins, their molecular diversity and their differential and combinatorial expression by neurons and glia (Shapiro and Colman, 1999; Yagi and Takeichi, 2000; Takeichi, 2007). Genetic analysis of the *Pcdh-α* and *Pcdh-γ* clusters in mice has uncovered phenotypes that are consistent with such roles, including disrupted dendrite arborization (Garrett et al., 2012; Lefebvre et al., 2012; Suo et al., 2012), impaired synaptic development (Weiner et al., 2005; Garrett and Weiner, 2009), mistargeting of axons (Hasegawa et al., 2008, 2012; Katori et al., 2009; Prasad and Weiner, 2011), and neuronal cell death (Wang et al., 2002b; Emond and Jontes, 2008; Lefebvre et al., 2008; Prasad et al., 2008; Chen et al., 2012). Similarly, recent work has implicated several Pcdh genes in a variety of human neurodevelopmental disorders (Redies et al., 2012). However, clear cellular functions, molecular mechanisms of protein interaction, and signaling partners have yet to be determined for several Pcdh subfamilies, and definitive evidence for Pcdh roles in synaptic recognition and adhesion does not yet exist. While much remains obscure about these fascinating cell-surface molecules, their demonstrated critical importance to neural development and their potential links to human disease suggest that further elucidation of the mechanisms regulating their expression, trafficking, interaction, and signaling will generate important new neurobiological insights.

In this review, we highlight selected recent results in several key areas of Pcdh biology and discuss their implications for our understanding of neural circuit formation and function. For comprehensive reviews of the Pcdh families see the following publications Yagi and Takeichi (2000), Redies et al. (2005, 2012), Morishita and Yagi (2007), and Kim et al. (2011).

## Clustered protocadherins: roles in neural circuit formation

Thus far, the best evidence in favor of a role for the clustered Pcdhs in neural circuit formation has been obtained for the *Pcdh-γ* family (Wang et al., 2002b; Weiner et al., 2005; Prasad et al., 2008; Garrett and Weiner, 2009; Prasad and Weiner, 2011; Chen et al., 2012; Garrett et al., 2012; Lefebvre et al., 2012; Suo et al., 2012). *Pcdh-γ* genes are expressed widely in the CNS, and γ-Pcdh proteins are found immunohistochemically at some, though far from all, synapses (Wang et al., 2002b; Phillips et al., 2003), as well as in dendrites, axons, and perisynaptic astrocytic processes (Garrett and Weiner, 2009). Mice in which all 22 *Pcdh-γ* genes (Wang et al., 2002b), or just the 3′-most variable exons (C-type exons; Chen et al., 2012) have been deleted lack voluntary movements and reflexes, and die shortly after birth. This phenotype is likely due to severe apoptosis and neurodegeneration of spinal interneurons and concomitant loss of synapses in the late embryonic period (Wang et al., 2002b; Prasad et al., 2008; Chen et al., 2012). A reduction in synaptic density is apparently a primary function of the γ-Pcdhs in the spinal cord, and not merely secondary to the observed neurodegeneration: When apoptosis is blocked in *Pcdh-γ* null mice by the additional loss of the pro-apoptotic gene *Bax* (Deckwerth et al., 1996), neuronal survival is rescued, but both excitatory and inhibitory synaptic puncta remain reduced by 40–50% and the double-mutant mice die at birth (Weiner et al., 2005). Further, when loss of the γ-Pcdhs is restricted to astrocytes, spinal cord synaptogenesis is delayed, but with no concomitant effect on apoptosis (Garrett and Weiner, 2009). Interestingly, mice lacking only the three C-type *Pcdh-γ* exons, in a *Bax*^−/−^ background, can survive past weaning, although they exhibit neurological impairments (Chen et al., 2012). This indicates that the loss of the C-type exons alone does not produce an exact phenocopy of the whole-cluster null.

In the retina, loss of all γ-Pcdhs also leads to apoptosis and synapse loss; however, in contrast to the spinal cord data, in this case blocking cell death by deletion of *Bax* does rescue synaptic density (Lefebvre et al., 2008). Conversely, when *Pcdh-γ* loss is restricted to the cerebral cortex, no excessive apoptosis is observed, but rather a major reduction in the dendritic arborization of cortical pyramidal neurons (Garrett et al., 2012). Together, these results from multiple regions of the CNS indicate that distinct neuronal types respond in different ways to the loss of the γ-Pcdhs. This is borne out by recent evidence that the γ-Pcdhs can mediate dendritic self-avoidance, in a manner very similar to that of the immunoglobulin superfamily molecule DSCAM (Fuerst et al., 2008, 2009), in both retinal starburst amacrine cells and cerebellar Purkinje neurons (Lefebvre et al., 2012). The dendritic phenotype (reduced arborization) observed in *Pcdh-γ* knockout cortical neurons (Garrett et al., 2012) and hippocampal neurons in which γ-Pcdh expression has been knocked down via RNAi (Suo et al., 2012) are not obviously consistent with such a self-avoidance role. Thus, it appears that the role of γ-Pcdhs in dendrite arborization may differ depending on the neuronal type, presumably due to a different repertoire of *cis*-interacting proteins and/or downstream signaling pathways.

The α-Pcdhs (originally termed Cadherin-related Neuronal Receptors, or CNRs) were the first of the clustered Pcdhs to be identified as synaptic molecules (Kohmura et al., 1998). The α-Pcdhs localize to developing axons (Blank et al., 2004; Morishita et al., 2004), consistent with the phenotypes subsequently observed in *Pcdh-α* mutant mice, which unlike the *Pcdh-γ* mutants are viable and fertile and do not exhibit increased neuronal apoptosis. Mice in which the *Pcdh-α* constant exons have been deleted exhibit an axonal targeting defect in the olfactory system, with axons expressing a given odorant receptor failing to coalesce on a single glomerulus in the olfactory bulb, as occurs in wildtype mice (Hasegawa et al., 2008, 2012). These disorganized axons appear to be able to form terminals and synapses at the glomeruli they contact; thus, the α-Pcdhs may be more important for axon guidance than they are for synaptogenesis (Hasegawa et al., 2008). Consistent with this, serotonergic axonal projections are also disorganized in mice lacking the α-Pcdhs, in some cases failing to penetrate the proper target area (Katori et al., 2009). Interestingly, morpholino knockdown of the *Pcdh-α* genes in zebrafish results in neuronal apoptosis, suggesting some distinct roles for the α-Pcdhs in different vertebrate systems (Emond and Jontes, 2008).

The *Pcdh-β* cluster remains the least studied of the three, perhaps because the lack of a shared constant domain among its members makes it more difficult to study. Using antibodies specific for two β-Pcdh proteins, β16 and β22, Junghans et al. (2008) found that both are present in the synaptic zones of the retina, the inner and outer plexiform layers, though only β16 was tightly localized to synapses, primarily the postsynaptic compartment. To date, no functional analysis of the *Pcdh-β* cluster has been published. However, Wu et al. (2007) have reported the generation of mice harboring large deletions within the clustered Pcdh loci, including loss of the *Pcdh-β* cluster, and presumably forthcoming analyses of such mice will yield functional data on the role of the β-Pcdhs in the nervous system.

## Clustered protocadherins: combinatorial complexity

The assumption that Pcdhs are *bona fide* cell adhesion molecules, acting in a manner analogous to that of the classical cadherins, is central to most views of Pcdh function (Shapiro and Colman, 1999; Redies et al., 2005; Takeichi, 2007). Though not essential for Pcdhs to play a role in neural circuit formation, differential homophilic cell adhesion is conceptually the most straightforward hypothesis, and there is some support for this. Multiple studies have shown that γ-Pcdhs can mediate homophilic interactions in a variety of cell types; the strength of adhesion, however, is modest in comparison to that of the classical cadherins (Frank et al., 2005; Fernandez-Monreal et al., 2009; Schreiner and Weiner, 2010). However, γ-Pcdhs overexpressed in heterologous cells do not efficiently reach the cell surface unless their cytoplasmic domains are truncated (Frank et al., 2005; Fernandez-Monreal et al., 2009; Schreiner and Weiner, 2010), suggesting that in some experiments “weak” adhesion may be due to low surface delivery of the molecules, which is typically not assessed. The α-Pcdhs have not yet been found to exhibit significant adhesive activity, suggesting that they do not act as homophilic cell adhesion molecules (Morishita et al., 2006), though some may exhibit heterophilic interactions with β1 integrins (Mutoh et al., 2004). However, only one (Pcdh-α4) of the 8 α-Pcdhs that contain an integrin-binding RGD site within EC1 has been tested for binding to integrins. Furthermore, the ubiquitously-expressed Pcdh-αC1 and -αC2 do not contain such an RGD site, making it unlikely that they interact with integrins. Therefore, the role of α-Pcdhs in cell adhesion remains uncertain.

**Figure 1:**
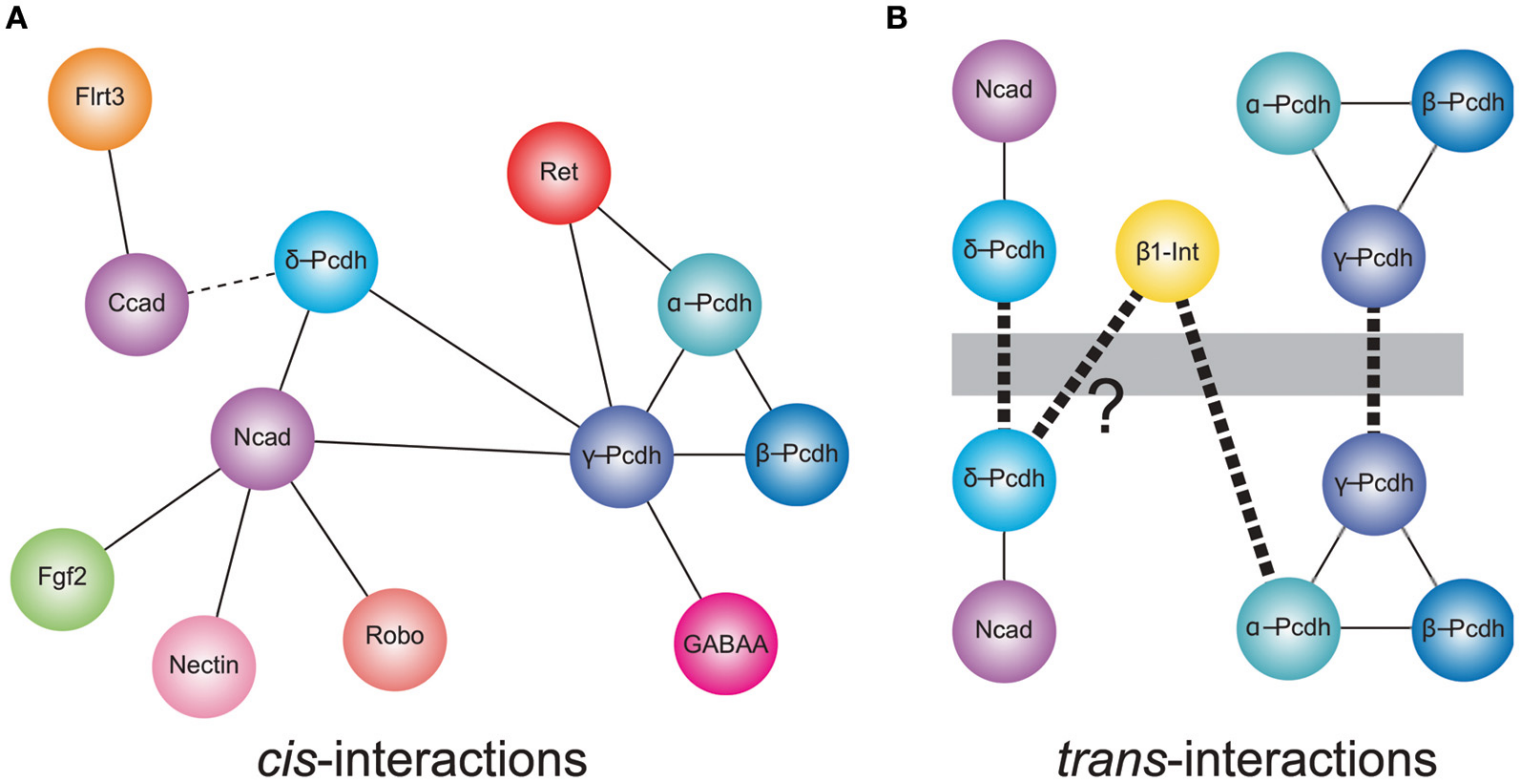
***Cis*- and *trans*-interactions of the protocadherins. (A)** Shown are cis-interactions at the membrane that have been identified for each of the protocadherin sub-families. Proteins containing cadherin repeats in their ectodomains (Ncad, α-, β-, γ- and δ-Pcdhs, and Ret) may mediate a core set of protein–protein interactions. These may be augmented by an expanded set of cis-interacting partners. In addition, δ-Pcdhs and γ-Pcdh isoforms can exist as homo- or hetero-oligomers, respectively. Lines represent direct interactions reported in the literature. The dashed line indicates that a direct interaction between C-cadherin and PAPC has not yet been demonstrated. **(B)** Members of each of δ-protocadherin and γ-protocadherin sub-families have been shown to mediate trans-homophilic interactions *in vitro*. These interactions occur in the context of larger macromolecular complexes. In the case of the δ2-protocadherin, Pcdh19, a Pcdh19-Ncad complex mediates *trans*-interactions. For γ-Pcdhs, heteromeric complexes of γ-Pcdh isoforms mediate trans-association *in vitro*. Proteomics studies also suggest that the clustered protocadherins exist in complexes that include α-Pcdh, β-Pcdh, and γ-Pcdh. Thus, the α-Pcdhs and β-Pcdhs could act as co-factors to modulate the γ-Pcdhs or could contribute to expanding the combinatorial complexity of cell interactions. In addition, mammalian α-Pcdhs contain an RGD sequence and exhibit trans-heterophilic interactions with β1-integrin. Similar RGD sequences are present in the δ2-Pcdhs, Pcdh17, and Pcdh19 suggesting that these proteins may also mediate heterophilic binding [indicated by (?)]. Heavy dashed line indicates *trans*-interactions.

Schreiner and Weiner (2010) recently provided the strongest evidence to date that any of the clustered Pcdhs can mediate homophilic interactions, while at the same time demonstrating that the rules governing these interactions are likely to be much more complex than had been assumed. Using a quantitative, colorimetric assay for cell adhesion, these authors confirm that γ-Pcdhs mediate homophilic interactions, but go on to show that these homophilic *trans-*interactions occur between heteromeric *cis* complexes that are most likely tetramers based on their size (Figure 1; Schreiner and Weiner, 2010). These tetramers are formed by the 22 γ-Pcdhs promiscuously, with no apparent isoform restriction. Thus, the maximal number of *cis-*tetramers that can form is 234,256 (22^4^), though assuming a degree of functional equivalence among tetramers with the same composition (that is, assuming topological organization within the membrane is not critical) the number is more likely to be on the order of 10^4^ (Zipursky and Sanes, 2010; Yagi, 2012).

In addition to cis-heteromers of γ-Pcdh isoforms, evidence suggests that α-Pcdh, β-Pcdh, and γ-Pcdh proteins also associate in complexes. A potential functional relationship between the α-Pcdhs and γ-Pcdhs was initially shown by Murata et al. (2004), who found that the γ-Pcdhs could facilitate trafficking of α-Pcdhs to the cell surface in transfected HEK293 cells, in which the latter rarely make it to the plasma membrane alone (Murata et al., 2004). In the absence of γ-Pcdhs, α-Pcdhs are retained in the endoplasmic reticulum and Golgi apparatus, with only low levels delivered to the cell surface (Murata et al., 2004). More recently, biochemical studies have demonstrated that α-, β-, and γ-Pcdhs can all be co-isolated by immunoprecipitation, both *in vitro* and *in vivo* (Chen et al., 2009a; Han et al., 2010; Biswas et al., 2012). The functional significance of such pan-cluster complexes remains unclear, but it is tempting to suggest that the α- and β-Pcdhs could modify the homophilic specificity exhibited by γ-Pcdh multimers. If so, the resulting combinatorial explosion in adhesive interfaces would mean, in theory, that the clustered Pcdhs have the ability to endow essentially every neuron with a unique molecular identity (Yagi, 2012).

## Protocadherin–cadherin interactions

There is increasing evidence for a relationship between the non-clustered δ-Pcdhs and classical cadherins (Figure 1). The Pcdh8-like molecule, PAPC (paraxial Pcdh), mediates cell sorting in Xenopus animal cap assays, which was initially taken as evidence for homophilic cell adhesion (Kim et al., 1998). More recently, Gumbiner and colleagues showed that PAPC does not itself mediate cell adhesion. Stable lines expressing PAPC failed to exhibit adhesion either in laminar flow assays or in cell aggregation assays (Chen and Gumbiner, 2006). Moreover, fusions of the PAPC ectodomain to the Fc region of IgG failed to mediate adhesion in bead aggregation assays (Chen and Gumbiner, 2006). Thus, despite the ability to effect cell sorting, PAPC does not function as a cell adhesion molecule. Chen and Gumbiner (2006) went on to resolve this apparent conflict by demonstrating that PAPC actually antagonizes adhesion by C-cadherin, although they did not demonstrate a physical interaction between the two proteins (Chen and Gumbiner, 2006). A similar antagonistic relationship was found between Pcdh8/Arcadlin and N-cadherin in cultured hippocampal neurons (Yasuda et al., 2007). Induction of Pcdh8 expression by electroconvulsive shock treatment in rats results in internalization of Ncad and removal from synaptic junctions. In this instance, there is a physical interaction of Pcdh8 with N-cadherin, as the proteins can be co-immunoprecipitated. Upon expression, Pcdh8 associates with Ncad, and *trans*-interactions mediated by Pcdh8 induce internalization through a pathway that involves the TAO2β kinase and p38 MAPK (Yasuda et al., 2007). Thus, there appears to be a close functional relationship between δ2-Pcdhs and classical cadherins.

More recently, Biswas et al. (2010) found that the δ2-Pcdh, Pcdh19, interacts with Ncad, both physically and functionally in the developing zebrafish. Knockdown of Pcdh19 in zebrafish embryos impairs neural plate convergence, resulting in malformation of the anterior neural tube. This phenotype is very similar to that of Ncad mutant embryos (Lele et al., 2002; Hong and Brewster, 2006; Biswas et al., 2010), suggesting that these molecules participate in a common pathway. Partial loss of both Pcdh19 and Ncad was shown to be synergistic, indicating that these two proteins cooperate during neural plate convergence. In addition to this functional interaction, Pcdh19 and Ncad associated physically to form a *cis*-complex (Biswas et al., 2010). In a follow up study, Emond et al. (2011) showed that secreted, epitope-tagged ectodomains of Pcdh19 and Ncad associate and can be purified from culture medium as a complex (Emond et al., 2011). When used in bead aggregation studies, this Pcdh19-Ncad complex mediates robust homophilic adhesion, although Pcdh19 on its own is not adhesive. Importantly, three lines of evidence support the idea that, within the complex, Pcdh19, rather than Ncad, is responsible for the adhesive interaction: (1) Pcdh19-Ncad complexes formed using adhesion-deficient Ncad mutants still mediate adhesion; (2) Mutations in Pcdh19 abolish adhesion by the complex; and (3) the Pcdh19-Ncad complex does not interact in *trans* with Ncad alone. Thus, Ncad appears to act as a cofactor to facilitate adhesive interactions of Pcdh19. Moreover, these results also indicate that Ncad is unavailable to mediate homophilic interactions when in complex with Pcdh19. These data suggest a model in which Ncad exists in one of two adhesive states: (1) Ncad directly mediates homophilic adhesion on its own; or (2) Ncad acts as a co-factor in *cis* to facilitate adhesion by Pcdh19 in *trans*. As another δ2-Pcdh, Pcdh17, exhibited similar behavior, Ncad may participate in multiple adhesive complexes with mutually incompatible specificities. Collectively, the data on PAPC, Pcdh8, Pcdh17, and Pcdh19 suggest that δ2-Pcdhs, at least, may affect cell adhesion (both positively and negatively) by forming complexes with classical cadherins.

There are hints that the participation of Pcdhs in homophilic interactions could be further complicated by the formation of larger macromolecular assemblies (Figure 1). In addition to a *cis*-interaction with δ-Pcdhs, Ncad also associates with other cell surface proteins, including Fgf receptor 2 (Williams et al., 2001), Nectin-2 (Morita et al., 2010), Cdo (Kang et al., 2003), and Robo (Rhee et al., 2002, 2007), and interacts functionally with β1-integrin (Arregui et al., 2000; Li et al., 2000). Moreover, C-cadherin, which interacts with PAPC, can also associate with the leucine-rich repeat protein, Flrt3 (Chen et al., 2009b). In addition, there is evidence that individual clustered Pcdhs or heteromeric complexes of α-, β-, and γ-Pcdhs can interact with classical cadherins (Ncad and Rcad) and δ-Pcdhs (Pcdh17) (Han et al., 2010), as well as Ret (Schalm et al., 2010) and GABA-A receptors (Li et al., 2012). Thus, the distinct cellular roles of clustered (α-Pcdh, β-Pcdh, and γ-Pcdh), non-clustered (δ-Pcdh), and classical cadherins may be difficult to define, due to the possibility of both crosstalk between and cooperation among the distinct family members. The association of Pcdhs with other gene families also suggests that a shifting protein composition within these complexes could influence the specificity of *trans*-interactions and the resulting downstream signaling pathways.

## Regulation of protocadherin trafficking

A key element in the regulation of many cell surface proteins is the control of their trafficking, and recent data suggest this especially to be true for Pcdhs. Initial work with γ-Pcdhs found that they are present largely in intracellular organelles, with a surprisingly low proportion present on the plasma membrane (Phillips et al., 2003; Murata et al., 2004). The control of γ-Pcdh trafficking appears to be largely dependent on elements within the cytoplasmic domain: deletion of either the constant domain, or the entire cytoplasmic domain, significantly increases surface delivery of the γ-Pcdhs (Fernandez-Monreal et al., 2009; Schreiner and Weiner, 2010). In addition, the receptor tyrosine kinase, Ret, may regulate the trafficking and stability of α-Pcdh/γ-Pcdh complexes through phosphorylation (Schalm et al., 2010). Ret was shown to associate with α- and γ-Pcdhs in a neural tumor cell line and to control their protein levels: knockdown of Ret resulted in a corresponding reduction in the levels of Pcdhs (Schalm et al., 2010). Interestingly, the ectodomain of Ret contains cadherin repeats, reinforcing the notion that cadherin EC repeats may be protein–protein modules that act as scaffolds to assemble *cis*-macromolecular assemblies. These results are reminiscent of the observations of Yasuda et al. (2007), which showed that the δ2-Pcdh Pcdh8/Arcadlin associates with Ncad to induce endocytosis and removal from synaptic junctions. Thus, complex formation may be a fundamental mechanism for regulating Pcdh localization, adhesion, trafficking, and stability.

While surface delivery of the γ-Pcdhs is regulated by their cytoplasmic domains (Fernandez-Monreal et al., 2009; Schreiner and Weiner, 2010), there is also evidence that the γ-Pcdhs themselves may regulate vesicular traffic in the cell. Using correlative light and electron microscopy (CLEM), Hanson et al. (2010) showed that overexpression of γ-Pcdhs, but not Ncad, in HEK293 cells leads to the formation of elaborate membrane tubules that appear to emanate from lysosomes. Intriguingly, tubules did not form when γ-Pcdh isoforms lacking a variable cytoplasmic domain were expressed (O'Leary et al., 2011), and the width of the tubules produced was reduced when half of the ectodomain was deleted (Hanson et al., 2010). While this last result provocatively suggests that homophilic γ-Pcdh ectodomain interactions could occur within intracellular organelles, the functional significance of the tubules formed by overexpression of γ-Pcdh cDNAs in heterologous cell lines remains unclear.

## Regulation of clustered protocadherin expression

**Figure 2:**
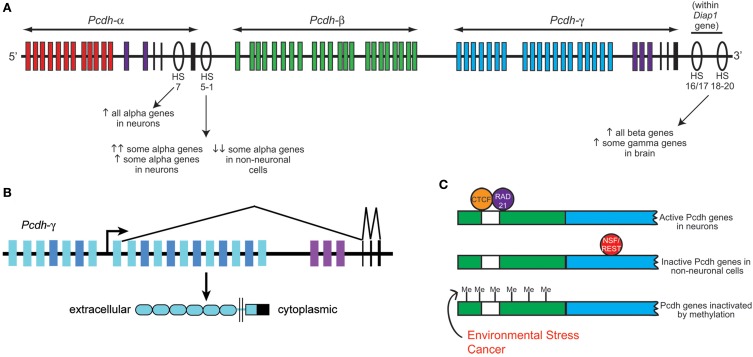
**Clustered protocadherin gene regulation. (A)** Schematic of the *Pcdh*-α, -β, and -γ clusters found in the mammalian genome. *Pcdh-α1-12* variable exons are shown in red, *Pcdh-β* genes in green, *Pcdh-γ* A and B subfamily variable exons in blue, and the homologous *Pcdh-α* and *-γ* C family variable exons in purple. *Pcdh-α* and *-γ* constant exons are shown in black. Several DNase I hypersensitive sites (HS) that have been identified as enhancers are shown as ovals, with their reported effects on clustered Pcdh gene expression noted below. Schematic shows approximate locations along the chromosome but is not to strictly to scale. **(B)** Schematic of the mouse *Pcdh-γ* cluster showing an example pattern of gene transcription and splicing. Each variable exon has its own upstream promoter from which transcription is initiated. A long transcript through the rest of the cluster is subsequently spliced such that each transcript contains only the 5′-most variable exon (which encodes the entire extracellular domain, the transmembrane domain, and a proximal cytoplasmic domain) and the three constant exons (which encode a further ~125 amino acid C-terminal cytoplasmic domain). *Pcdh-α* transcription and splicing occurs similarly. **(C)** Schematics of a typical clustered Pcdh promoter region (green) containing the conserved sequence element (CSE; white) and an adjacent variable exon (blue). In neurons, CTCF and Rad21 bind near the CSE and promote expression in concert with the HS5-1 enhancer element. In non-neuronal cells, NSF/REST may suppress gene expression by binding to canonical and non-canonical NRSE sites either in the promoters (*Fugu*) or within the coding sequences (mammals). Hypermethylation of clustered Pcdh promoters may also inactivate gene expression; increased methylation has been reported in various cancerous cell types and in brain following environmental stressors such as poor maternal care.

One of the most fascinating aspects of the clustered Pcdh families is their differential and combinatorial expression in cells of the nervous system. A decade ago, Tasic et al. (2002) and Wang et al. (2002a) concurrently identified the mechanism of *Pcdh-α* and *-γ* transcription. The sequence upstream of each variable (V) exon contains its own promoter region including a ~20 base pair conserved sequence element (CSE) that is required for expression. Through mechanisms that are still not entirely clear, a given V exon promoter is “chosen” and transcription through the remaining portion of the *Pcdh-α* or -γ cluster proceeds; intervening V exons are then removed when the 5′ V exon is *cis-*spliced to the three downstream constant (C) exons (Tasic et al., 2002; Wang et al., 2002a) (Figure 2). Although the *Pcdh-β* cluster does not contain its own C exons, each *Pcdh-β* V exon harbors a consensus 5′ splice site near its end (Wu and Maniatis, 1999) suggesting the possibility of splicing to the C exons of other clusters. Such intercluster spliced transcripts, while rare, are apparently present in neurons, as are low levels of αV/γC and γV/αC hybrid transcripts produced by *trans*-splicing between separate pre-mRNA intermediates (Tasic et al., 2002; Wang et al., 2002a). Most of the *Pcdh-α, -β*, and -γ V exons are expressed monoallelically; though both cluster alleles are transcriptionally active in a given cell, an individual V exon promoter is only “chosen” from one of the two alleles (Esumi et al., 2005; Kaneko et al., 2006). The exception to this rule are the nearly ubiquitously-expressed *Pcdh-αC1* and *αC2* V exons, and the related *Pcdh-γ C3*, *C4*, and *C5* V exons, all of which can be biallelically expressed (Esumi et al., 2005; Kaneko et al., 2006). Single-cell RT-PCR analysis of cerebellar Purkinje cell neurons suggests that each cell expresses ~4 *Pcdh-α* isoforms (2 of the monoallelically expressed genes plus the 2 ubiquitous 3′ genes), ~2 *Pcdh-β* isoforms and ~7 *Pcdh-γ* isoforms (~4 of the monoallelically expressed genes plus the 3 ubiquitous 3′ genes) (Hirano et al., 2012; Yagi, 2012).

A key insight into the control of clustered *Pcdh* expression came with the discovery by Ribich et al. (2006) of two long-range regulatory elements located near the 3′ end of the *Pcdh-α* cluster. These sites were identified based on sequence conservation and hypersensitivity to DNase I degradation; hypersensitive sites (HS) 5-1 lie 3′ of the third α constant exon, and HS7 lies between α constant exon 2 and 3 (Figure 2). Addition of HS5-1 (or to a much lesser extent, HS7) downstream of a minimal promoter-*LacZ* cassette resulted in reporter expression throughout the CNS, demonstrating that this site can independently promote gene expression in regions known to express the *Pcdh-α* genes (Ribich et al., 2006). Deletion of the HS5-1 site in mice led to significantly reduced expression of most *Pcdh-α* genes: Expression of the 5′ V exons, *Pcdh-α1-5*, was moderately reduced, while that of the more 3′ *Pcdh-α6-12* and -*αC1* were greatly reduced (Kehayova et al., 2011; Yokota et al., 2011). The *Pcdh-αC2* gene was unaffected by HS5-1 deletion, consistent with prior *in vitro* results (Ribich et al., 2006). Deletion of the HS7 enhancer site resulted in a more moderate, but more uniform, negative effect on *Pcdh-α* gene expression, with expression of all V exons significantly reduced in the cerebellum (Kehayova et al., 2011). Further HS sites downstream of the *Pcdh-γ* cluster were identified and termed HS16-20 by Yokota et al. (2011), who showed that deletion of these sites in mice led to a nearly complete loss of expression across the *Pcdh-β* cluster. Surprisingly, deletion of HS16-20 had a much less drastic effect on the more closely adjacent *Pcdh-γ* cluster; no effect was found on genes of the *Pcdh-α* cluster in these mice (Yokota et al., 2011).

The identification of these long-range regulatory sites provides a likely explanation for the results of Noguchi et al. (2009), who generated several lines of mice harboring deletions or duplications within the *Pcdh-α* cluster using targeted meiotic recombination. Across 4 lines of mice (deletion of α11-C2; deletion of α2-11; duplication of α2-10; duplication of α12-C2), the total *Pcdh-α* transcript levels, as measured by assaying the shared constant exons, remained fairly consistent (Noguchi et al., 2009). In the deletion lines, the remaining exons were upregulated, while in the duplication lines, individual exons were downregulated, to maintain expression levels. Consistently, the 3′-most V exon in each line took on the ubiquitous expression pattern normally found only for *Pcdh-αC1* and -α*C2* (Noguchi et al., 2009). This suggests that proximity to the HS5-1 and/or HS7 sites, in part, regulates expression levels of individual exons and may account for the varying expression levels across the *Pcdh-α* cluster.

Two zinc finger transcription factors recently have been identified to regulate the clustered *Pcdh* genes: neuron-restrictive silencer factor/RE-1 silencing transcription factor (NRSF/REST), and CTCF (CCCTC-binding factor). NRSF/REST binds to neuron-restrictive silencer elements (NRSEs) to repress neuronal gene expression in non-neuronal cells (Chong et al., 1995; Schoenherr and Anderson, 1995). There are multiple canonical and non-canonical NRSF-binding sites (NRSEs) within the fugu (pufferfish), mouse, and human *Pcdh* clusters (Tan et al., 2010), and deletion of NRSEs from *Pcdh* constructs causes their expression in transgenic *Xenopus* tadpoles to shift from neural-specific to ubiquitous. Kehayova et al. (2011) confirm that the HS5-1 element contains a non-canonical NRSE site, and that this site is required for the suppression of mouse *Pcdh-α* promoter activity in a kidney cell line.

CTCF binding sites are found within the promoters of *Pcdh-α* V exons, as well as within HS5-1 (Kehayova et al., 2011; Golan-Mashiach et al., 2012). This is particularly interesting, as CTCF is known to mediate enhancer/promoter interactions through DNA looping (Gillen and Harris, 2011). Several experimental observations are consistent with such a role at the *Pcdh-α* locus: (1) Knockdown of CTCF with siRNAs reduces the expression of 2 assayed *Pcdh-α* genes in the HEC1-B cell line (Golan-Mashiach et al., 2012); (2) As assayed by chromatin immunoprecipitation (ChIP) from brain samples, deletion of HS5-1 leads to reduced CTCF binding at those *Pcdh-α* promoters that are strongly affected by HS5-1 deletion, but not at those that are only weakly affected (Kehayova et al., 2011); and (3) Transcriptionally active *Pcdh-α* promoters bind both CTCF and the nuclear phosphoprotein Rad21, a subunit of the cohesin complex involved in sister chromatid cohesion during mitosis. The HS5-1 enhancer also binds CTCF and Rad21, and knockdown of either CTCF or Rad21 reduces the expression of several alternatively-expressed *Pcdh-α* isoforms (Monahan et al., 2012). It will be important in the future to determine whether similar roles for NRSF/REST, CTCF, and cohesin exist at the *Pcdh-γ* cluster, and to uncover the mechanisms by which these factors collaborate to render a “promoter choice” within individual neurons.

A spate of recent papers has revealed that the clustered Pcdh genes can be epigenetically silenced by methylation, and that dysregulation of this process may underlie multiple types of cancer as well as the deleterious effects of environmental stress. Using two mouse cell lines, Kawaguchi et al. (2008) showed that methylation of each *Pcdh-α* promoter, as well as the 5′ region of each V exon, correlates negatively with its expression level; experimentally inducing demethylation increased *Pcdh-α* transcription, while inducing hyper methylation decreased it. Consistent with their ubiquitous expression in neurons, the promoters of *Pcdh-αC1* and *-αC2* are hypomethylated *in vivo* (Kawaguchi et al., 2008). Studies have found that hypermethylation of CpG islands (CGIs) within *Pcdh-α* and -*β* variable exons was highly predictive of poor prognosis across a large group of neuroblastoma samples (Abe et al., 2005). However, this increased methylation does *not* result in the decreased expression of *Pcdh-β* genes in tumor samples, indicating that while *Pcdh* hypermethylation is statistically predictive of cancer outcomes, the underlying mechanism does not involve *Pcdh* expression levels *per se* (Abe et al., 2005).

A more direct link between the clustered *Pcdhs* and cancer progression has been forged by studies from Karim Malik and colleagues (Dallosso et al., 2009, 2012). Genome-wide analysis of promoter hypermethylation in Wilms' tumor (WT), a pediatric cancer of the kidney, identified the three *Pcdh* clusters in patient samples. Multiple *Pcdh-α*, *-β*, and *-γ* genes were found to be hypermethylated in WT samples; while *Pcdh-α* gene expression is not normally detectable in fetal kidney, *Pcdh-β* and *-γ* expression is, and the latter is consistently downregulated in WT, with some genes ~90% silenced (Dallosso et al., 2009). Importantly, siRNA knockdown of *Pcdh-γ* genes in kidney cell lines leads to increased β-catenin/TCF reporter gene activity and increased expression of known target genes of the canonical Wnt pathway, which is known to be constitutively active in WT. Conversely, overexpression of *Pcdh-γ* cDNAs in WT and HEK293 cell lines leads to growth inhibition in soft agar assays (Dallosso et al., 2009). More recently, a tumor suppressor function was confirmed specifically for *Pcdh-γC3* in colorectal adenoma and carcinoma cells: overexpression of constructs encoding γ-Pcdh-C3 suppresses Wnt and mTOR signaling and reduces colony formation in the colon carcinoma cell line HCT116 (Dallosso et al., 2012).

Together, these exciting results suggest that Pcdhs may be an important new therapeutic target in multiple types of cancer. Importantly, several of the classical cadherins have been implicated in cancer progression; for example, loss of E-cad is a common event in a variety of epithelial cancers (Cavallaro and Christofori, 2004). The demonstrated interaction between classical cadherins and members of the Pcdh family, therefore, suggests that modulation of Pcdh expression or localization could have follow-on effects on the classical cadherins that could regulate tumor progression. It will be important in future studies to re-assess the vast literature on classical cadherins in cancer in light of their demonstrated regulation by Pcdhs, as well as to determine whether Pcdh expression is disrupted in various tumor cell types.

The clustered Pcdhs may also play a role in the brain's response to environmental stress, based on studies showing that poor maternal care [assessed by the frequency of maternal licking and grooming (LG) behaviors] correlates with changes in the methylation of their genes. McGowan et al. (2011) analyzed methylation, histone H3-lysine-9 (H3K9) acetylation, and gene expression patterns across 7 MB surrounding the gene encoding the glucocorticoid receptor NR3C1, a prominent target for mediating response to stress, in hippocampal samples from offspring of rat mothers who exhibited high or low LG (McGowan et al., 2011). The three Pcdh gene clusters are located within this region on rat chromosome 18, and hypermethylation of multiple Pcdh genes was observed in offspring of low LG mothers. Conversely, many of the genes across the three Pcdh clusters show significantly higher expression in offspring of high LG mothers (McGowan et al., 2011). A follow-up study showed that this pattern is conserved in humans: hippocampal samples from suicide completers with a history of severe child abuse exhibited hypermethylation across the *Pcdh* gene clusters (Suderman et al., 2012). These epigenetic studies are particularly compelling given the dependence of proper serotonergic axon targeting on the α-Pcdhs (Katori et al., 2009), and of cortical dendrite arborization on the γ-Pcdhs (Garrett et al., 2012), and indicate that the clustered Pcdhs may be critical mediators of neural circuit changes in response to environmental stress during brain development.

## Conclusions

The standard, *a priori* view of Pcdhs was that they simply represented an expanded complement of classical cadherin-like cell adhesion molecules. Accumulating evidence suggests that this model is incomplete. From the recent literature, several insights into Pcdhs can be gleaned. First, Pcdhs appear to function as part of larger macromolecular assemblies. The clustered Pcdhs form heteromeric *cis*-complexes that include α-, β-, and γ-Pcdhs, as well as Ret kinase, and, potentially, many other proteins (Chen et al., 2009a; Han et al., 2010; Schalm et al., 2010; Schreiner and Weiner, 2010). In addition, δ2-Pcdhs can physically associate with classical cadherins, and the Pcdh-Cad complexes appear to be important *in vivo* (Yasuda et al., 2007; Biswas et al., 2010; Emond et al., 2011). As Ret also contains cadherin repeats in its ectodomain, these results could suggest that the cadherin motif functions as a mediator of protein–protein interactions to assemble multiprotein *cis*-complexes, in addition to their known role in homophilic *trans*-interactions. Second, the role of Pcdhs in cell adhesion can be complex and indirect. Some Pcdhs may be able to act as adhesion molecules on their own, others appear to mediate adhesion only by associating with one or more cofactors or coreceptors, while still others clearly inhibit cell adhesion by antagonizing classical cadherin interactions.

When considered broadly, the recent data suggest two significant concerns: (1) Adhesion studies in reduced *in vitro* systems (including those based in heterologous cells and those utilizing purified proteins on beads or substrates) may not recapitulate the nature of adhesive interactions as they would occur in endogenously-expressing cell types *in vivo*. Thus, the individual ectodomains used in bead-based assays or the expression of molecules in heterologous cells likely will not reflect the adhesive interactions of multimolecular protein assemblies occurring *in vivo*. In the future, this should be addressed by better defining the complexes that exist *in vivo*, which would then allow for a more realistic reconstitution of complexes for *in vitro* studies. (2) Interpreting functional experiments, such as knockouts, as demonstrating a specific role for a molecule *per se* should be seen as provisional, as further work is needed to show whether the observed phenotype is due specifically and directly to the loss of the targeted molecule, or to shifts in the composition and function of multi-protein assemblies that may vary with cell-type and developmental time. It will, thus, be important to consider carefully the results of biochemical and proteomic experiments in reduced systems when interpreting functional studies, and to develop new strategies for dissecting this biological complexity (e.g., looking for genetic interactions in animals heterozygous for both a Pcdh and a known interactor). The recent biochemical experiments (e.g., Schreiner and Weiner, 2010; Emond et al., 2011) that have elucidated molecular mechanisms of Pcdh interactions have actually shown that it may be more difficult than initially believed to understand the cellular and developmental processes in which these molecules participate. With this in mind, we can likely look forward to many surprises as further studies of these far-from-prototypical adhesion molecules emerge.

### Conflict of interest statement

The authors declare that the research was conducted in the absence of any commercial or financial relationships that could be construed as a potential conflict of interest.
